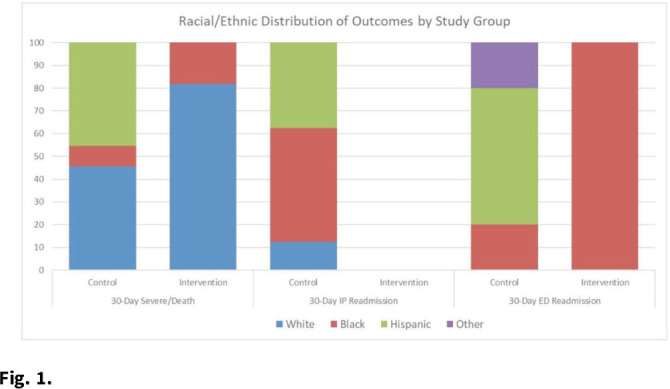# Care innovations and health disparities: An exploration of COVID-19 outcomes in inpatient and hospital-at-home care settings

**DOI:** 10.1017/ash.2022.138

**Published:** 2022-05-16

**Authors:** Jennifer Priem, Lisa Krinner, Stephanie Murphy, Sveta Monahan, Colleen Hole

## Abstract

**Background:** Hospital at home (HaH) programs have been a critical resource for providing inpatient care to acutely ill patients throughout the COVID-19 pandemic. Given that this innovative care delivery model relies on technology and environmental concerns, questions have been raised about the effectiveness of HaH for vulnerable groups. However, evidence is extremely limited regarding equity issues in the HaH context. Thus, we explored COVID-19 outcomes within vulnerable groups. **Methods:** We conducted a matched, retrospective study of 116 acutely ill patients with COVID-19, aged ≥18 years, who presented to an AH emergency department (ED) and were admitted for inpatient care. Treatment patients were admitted to AH HaH between July 15 and September 31, 2020, and control patients were hospitalized between May 8 and June 25, 2020. Patients were matched based on oxygen requirement and DS CRB-65 (DEFINE) score. Race or ethnicity and area deprivation index (ADI) were chosen as predictors of health disparities. The ADI incorporates 17 indicators of poverty, educational attainment, and housing quality at the census tract level. Outcomes included 30-day (from discharge) severe illness or death composite, IP readmission, and ED visit. **Results:** The frequency of 30-day severe illness or death and ED visits were equivalent between the groups (n = 11; ED n = 5); the proportion of severe illness was higher for White patients in AH-HaH (n = 9 vs n = 5), and for Hispanic patients treated in the hospital (n = 5 vs n = 0; Fig. 1). There were no 30-day inpatient readmissions in the AH-HaH group, but 8 readmissions occurred with inpatients. The distribution of severe illness among the ADI quintiles varied. For traditional inpatients, disease progression was limited to ADI Q3–5 (Q3 = 3, Q4 = 6, Q5 = 2); for AH-HaH patients, disease progression was not influenced by ADI. The effect of ADI on 30-day ED readmission was nonsignificant. **Conclusions:** Although exploratory in nature, the results suggest that HaH may help combat sources of health disparities that have dominated the pandemic. Although inpatient care resulted in inpatient readmissions, mainly among Black and Hispanic patients, AH-HaH stays were not associated with any inpatient readmissions. The equivalent distribution among ADI quintiles of patients who became severely ill within 30 days of their AH-HaH stay suggests that HaH may be able to leverage innovation to reach vulnerable populations and reduce the impact of factors that contribute to inequity.

**Funding:** None

**Disclosures:** None

**Figure:**